# Cultural Influences on Name Agreement: A Set of 237 Standardized Photographs of French Speakers in Quebec and France

**DOI:** 10.5334/joc.509

**Published:** 2026-07-06

**Authors:** Emie Lefebvre, Roxanne Turcotte, Ludovic Ferrand, Edith Durand

**Affiliations:** 1 Université du Québec à Trois-Rivières, Laboratoire SNC (Sensorimotricité, Neuroplasticité et Communication), CA; 2 Université du Québec à Trois-Rivières, Laboratoire SNC, CA; 3 CNRS, Université Clermont Auvergne, LAPSCO, Clermont-Ferrand, FR

**Keywords:** picture naming, name agreement, h-value, norms, French varieties comparison

## Abstract

This study investigated name agreement for a set of 237 photographs from the Bank of Standardized Stimuli (BOSS; Brodeur et al., 2010, 2014), using modal name agreement as a percentage-based measure of naming consensus and the H-value as an information-based measure of response dispersion. Data were collected through an online questionnaire completed by 105 Quebec French speakers and 106 France French speakers, who reported no history of neurological or psychiatric disorders. We hypothesized that cultural context would yield differences in H-values and modal names between the two groups of speakers representing two varieties of French. For each photograph, we report the dominant (modal) name, modal name agreement, alternative responses, and the corresponding H-value. Across the dataset, mean modal name agreement was 83.81% for Quebec French speakers and 83.34% for France French speakers, while mean H-values were 0.67 and 0.73, respectively. Distributional comparisons did not reveal significant differences in H-values between varieties. However, complementary item-level analyses indicated that H-values were slightly but significantly higher in France French, with a small effect size, and that H-values were positively associated across varieties. Descriptive comparisons also showed substantial overlap in modal names across varieties, while identifying a subset of items with different dominant labels. This study provides the first cross-cultural normative dataset for Quebec and France French, underscoring the importance of culturally adapted naming norms in both cognitive research and clinical practice.

## Introduction

Name agreement is a robust predictor of picture-naming performance and is known to vary across cultural and linguistic communities (Alario & Ferrand, 1999; Brodeur et al., 2012; Duñabeitia et al., 2018; Nishimoto et al., 2005; Sirois et al., 2006), making culturally grounded norming studies essential for understanding how speakers converge on lexical labels for visual stimuli. Name agreement refers to the extent to which different speakers agree on the same lexical label for a given picture. In most norming studies, name agreement is defined as the highest percentage of participants who produce the same label for a picture (Alario & Ferrand, 1999; Brodeur et al., 2014). Pictures that elicit several competing labels therefore show lower name agreement than pictures that are almost unanimously associated with a single name (Alario & Ferrand, 1999). For example, in the French norms reported by Alario and Ferrand (1999), some items showed very high name agreement, such as *accordion, shark* and *banana* (*H* = 0.00, 100% agreement), whereas others showed lower agreement, such as *bread* (*H* = 1.34, 46% agreement), *barn* (*H* = 1.12, 50% agreement), and *anteater* (*H* = 1.78, 21% agreement). Vitkovitch and Tyrrell (1995) further showed that low name agreement can arise from several sources, including shortened or elaborated labels, multiple correct names, and incorrect competitors from the same semantic category. In their British norms, for instance, *television* elicited labels such as *television*, *TV*, and *TV set* (64% agreement), whereas *watch* elicited both *watch* and *wristwatch* (84% agreement) (Vitkovitch & Tyrrell, 1995). Other items reflected competition between multiple correct names, as in the case of *sofa*, *settee*, and *couch* (69% agreement), or incorrect competitors, as when *ant* was named *spider* or *insect* by some participants (76% agreement) (Vitkovitch & Tyrrell, 1995).

Name agreement has been shown to influence both naming speed and accuracy: pictures with a single dominant name tend to be named faster and more accurately than pictures that elicit many alternative responses (Alario et al., 2004; Barry et al., 1997; Lachman et al., 1974; Vitkovitch & Tyrrell, 1995). Importantly, this effect holds even when controlling other variables such as age of acquisition and word frequency (Lachman et al., 1974; Randolph et al., 1999). As a result, name agreement is commonly measured as part of the norming process for pictorial stimuli such as photographs and line drawings (Brodeur et al., 2010). Name agreement can be quantified in several ways. Percentage name agreement, or modal name agreement, provides a direct measure of naming outcome consensus by indicating the proportion of participants who produced the most frequent name for a picture. The H-value, by contrast, is an information-based statistic that reflects how responses are distributed across different names (Shannon & Weaver, 1949; Snodgrass & Vanderwart, 1980). When all participants assign the same name to a given item, the H-value is equal to 0, corresponding to no response dispersion and maximal name agreement (Duñabeitia et al., 2018). Thus, higher H-values indicate greater dispersion in the names assigned to an image, and therefore lower name agreement (Brodeur et al., 2014; Duñabeitia et al., 2018). Unlike percentage agreement, the H-value reflects not only how many different names are produced for a given image, but also how strongly responses are concentrated on the most frequent label (Brodeur et al., 2014; Snodgrass & Vanderwart, 1980).

Based on the available literature, most existing name agreement norms in French have been collected using line drawings. Several studies have provided France French norms for sets of black-and-white line drawings, including name agreement (Alario & Ferrand, 1999; Bonin et al., 2003; Schwitter et al., 2004). French Canadian norms have also been reported for a comparable set of drawings (Sirois et al., 2006). Color line drawings have also been normed in large databases, including MultiPic, which comprises 750 pictures standardized in six European languages, including France French (Duñabeitia et al., 2018), and IMABASE, which comprises 313 pictures standardized in France French (Bonin et al., 2020). In contrast, fewer standardized photographic resources with French name agreement norms are available. However, photographic stimuli offer several advantages for both experimental research and clinical applications. For instance, photographs provide more realistic depictions of everyday objects and situations, and thus more closely approximate the visual input encountered in daily life (Brodeur et al., 2014). Furthermore, photographs have been argued to engage neural circuits that are similar to those recruited during real-world object processing (Brodeur et al., 2014). Photographs provide richer visual information than line drawings, including cues related to color, luminance, texture, and shading (Heuer, 2016). These additional cues can facilitate picture recognition in adults (Heuer, 2016) and may affect how the target stimulus is encoded and remembered (Biederman & Ju, 1988; Brodie et al., 1991; Leder, 1999; Rhodes et al., 1987). Several photographic databases have been developed in different linguistic or national contexts, including a set of action photographs developed in the United States (Fiez & Tranel, 1997), the International Affective Picture System, also developed in the United States (Bradley & Lang, 2007), and a set of ecological object photographs normed in Italy with Italian undergraduate students and English-speaking undergraduate students temporarily living in Florence for study or research programs (Viggiano et al., 2004). Despite the existence of these photographic databases, available French norms for photographic materials remain more limited than those available for line drawings. The Bank of Standardized Stimuli (BOSS; Brodeur et al., 2010, 2014) contains 1,468 photographs and, to our knowledge, is among the largest freely available picture databases for research (Brodeur et al., 2014). For French, available name agreement norms for BOSS photographs remain limited. Brodeur et al. (2012) provided Quebec French name agreement norms for 480 photographs based on data from 30 neurologically healthy adults, and Masson-Trottier et al. (2017) validated 235 photographs with 13 neurologically healthy Quebec French speakers. There appear to be no name agreement norms for BOSS photographs in France French. Establishing name agreement norms for BOSS photographs in both Quebec French and France French would therefore provide a valuable resource for studies that target French-speaking populations.

France French and Quebec French are often described as two national varieties of the same language, each associated with its own territory and sociocultural context (Reinke & Ostiguy, 2016). Speakers of different national varieties do not always use the same words to refer to the same objects or situations (Reinke & Ostiguy, 2016). Speakers from France and Quebec may therefore name the same picture differently, despite sharing a common base vocabulary. More broadly, cultural and linguistic factors have been shown to influence how pictures are recognized, evaluated, and named (Bonin et al., 2020; Duñabeitia et al., 2018). Several studies have reported that normative data collected in one cultural context do not always generalize to other populations, even when the same language is involved (Brodeur et al., 2010, 2012). For instance, some objects that are frequent and familiar in one country may be relatively unfamiliar in another and are sometimes removed from picture sets for this reason (Alario & Ferrand, 1999; Rossion & Pourtois, 2004). In the French norms reported by Alario and Ferrand (1999), items selected in an American context, such as a baseball bat, a football helmet, or a pretzel, were replaced by objects considered more common in the experience of French speakers. Cross-cultural comparisons have also shown reduced correlations in name agreement across cultures (Alario & Ferrand, 1999; Nishimoto et al., 2005; Sirois et al., 2006). In MultiPic, Duñabeitia et al. (2018) reported that a shared language increases but does not guarantee convergence in name agreement across countries. Notably, this database included separate Dutch norms for speakers from Belgium and the Netherlands, illustrating that national varieties of the same language may warrant distinct normative datasets (Duñabeitia et al., 2018). In addition, Brodeur et al. (2012) noted a descriptive difference in mean modal name agreement between American and British English norms. Although this difference was not statistically tested, mean modal name agreement was lower in the American norms reported by Cycowicz et al. (1997) than in the British norms reported by Barry et al. (1997), with values of 86% and 93%, respectively. This example suggests that cultural context can modulate naming patterns even among speakers of the same language. These findings suggest that picture naming norms may need to be adapted not only across languages, but also across national varieties of the same language.

Therefore, comparing France French and Quebec French norms for the same set of photographs is particularly informative. The present study aims to measure name agreement for 237 BOSS photographs in both Quebec French and France French. For each item and each group, we report the dominant (modal) name, alternative responses, modal name agreement, and the corresponding H-value as an information-based index of response dispersion. We then compare H-values and modal names between the two varieties to assess the extent to which cultural context shapes name agreement within French. The resulting norms are intended to provide a shared, cross-cultural reference for experimental and clinical studies that target adult French-speaking populations.

## Method

The study was approved by the Research Ethics Boards of the Université de Montpellier (CER-2023-049bis) and of the Université du Québec à Trois-Rivières (CER-23-303-07.06), and all participants provided informed consent online.

### Participants

A total of 211 adult French speakers took part in the study: 105 Quebec French and 106 France French speakers. The Quebec French group (mean age = 40 years) included 73 women, 30 men and 2 non-binary participants. For the France French group, complete demographic information (age and gender) was available for 44 participants (mean age = 52 years; 30 women and 14 men). For the remainder, only age-range information was available, but all were 18 years or older. Information about education level was available only for the Quebec French sample. Education level was grouped according to the Quebec education system, in which 11 years corresponds to completed secondary education and 16 years corresponds to a completed bachelor’s degree. In this group, 16 participants reported fewer than 11 years of education (15.2%), 54 reported 11 to 15 years (51.4%), and 35 reported 16 years or more (33.3%).

Participants were recruited through social media. All reported French as their native language, corresponding to the local variety (Quebec French in Quebec, France French in France). Inclusion criteria were being at least 18 years old, having no history of neurological or psychiatric disorders, and having no uncorrected visual impairments. No further age restrictions were imposed. According to available data, the oldest participant was 82 years old in the France French group and 85 years old in the Quebec French group.

### Materials

The stimuli were 237 color photographs depicting common concrete objects. Selection was based on the standardized set of 260 black-and-white line drawings by Snodgrass and Vanderwart (1980), which is widely used in picture naming research and has been normed in several languages (Duñabeitia et al., 2018). Snodgrass and Vanderwart classified 189 items into semantic categories, and this classification was retained. For the remaining 71 items, which had no original category, we used the semantic categories proposed in IMABASE (Bonin et al., 2020) when the item was present there and grouped the remaining concepts into a residual category. BOSS did not contain photographs for 37 concepts from the Snodgrass and Vanderwart set. When more than 20% of the items in the semantic category were missing, replacement photographs were selected from online repositories under a Creative Commons license. Categories with fewer missing items were simply reduced. This procedure resulted in a final set of 237 photographs, including 223 from BOSS and 14 replacement photographs obtained under Creative Commons licenses.

### Procedure

The naming task was administered online in four data collection waves, first in France and then in Quebec. In France, data were collected from September to October 2021 (*n* = 62) and from April to June 2023 (*n* = 44). In Quebec, data were collected from February to March 2024 (*n* = 53) and from June to August 2024 (*n* = 52). Google Forms was used for the first three waves, and LimeSurvey was used for the fourth wave.

The online questionnaire was refined across data collection waves, while the core picture-naming task remained the same. After the first France French wave, the questionnaire was modified to collect participants’ gender and exact age, whereas only age ranges had been collected initially. For the Quebec French waves, additional demographic questions were added, including education level and languages spoken by participants. Following an ethics-approved modification to the protocol, the fourth wave was administered with LimeSurvey. This platform allowed item order to be randomized for each participant, enabled participants to pause while completing the questionnaire, recorded time spent in the questionnaire, provided a more flexible question format, and did not require participants to use a Google account.

Across all waves, participants first provided informed consent online and completed a short demographic questionnaire before starting the picture-naming task. Each trial consisted of one color photograph presented with an open response field. Participants were instructed in French to identify the object as briefly and unambiguously as possible by writing the first name that came to mind, preferably as a single word when possible. No time limit was imposed, and no feedback was provided. In the Google Forms versions, the 237 photographs were presented in a fixed order based on the sequence proposed by Snodgrass and Vanderwart (1980) and organized by semantic category. In the LimeSurvey version, the same 237 photographs were presented in a randomized order for each participant. For the LimeSurvey wave, time spent on the picture-naming task was also recorded. The median recorded completion time for picture naming was 28.4 min (*M* = 36.4 min, *SD* = 35.5 min), although completion time varied substantially across participants.

### Data Processing

A data processing procedure was implemented to prepare the responses for automated computation of the H-value and extraction of the modal name and alternative responses. The full data processing procedure is documented in Supplementary File A, available in the Borealis repository. Processing decisions were based on previous norming studies on name agreement (Bonin et al., 2020; Brodeur et al., 2014; Decuyper et al., 2021; Duñabeitia et al., 2018; Martínez et al., 2020; Snodgrass & Vanderwart, 1980). Whenever an unfamiliar label was written, we consulted Usito, a dictionary describing standard French as used in Quebec (Université de Sherbrooke, n.d.), and the Vitrine linguistique of the Office québécois de la langue française (Gouvernement du Québec, 2025). For both Quebec French and France French datasets, all modifications to the raw responses were documented in dedicated data processing logs for internal quality control. An initial processing pass was carried out by one researcher, and all entries were subsequently checked by a research assistant to minimize errors.

### Analyses

From the processed datasets, we computed, for each of the 237 photographs and for each variety, modal name agreement, defined as the percentage of participants who produced the most frequent name, and the H-value, an information-based index of response dispersion across naming alternatives. The H-value was calculated as:


$$H=\overset{k}{\underset{i=1}{\Sigma }}P_{i}{\text{log}}_{2}\left(\frac{1}{P_{i}}\right)$$

In this expression, *k* is the number of distinct names produced for a given picture, and *P_i_* is the proportion of participants who produced the *i*-th name (Brodeur et al., 2014). A picture for which all participants produce the same label yields an H-value of 0, which corresponds to no response dispersion and maximal name agreement (Duñabeitia et al., 2018). Higher H-values indicate greater response dispersion and therefore lower name agreement (Brodeur et al., 2014; Duñabeitia et al., 2018). Responses left blank or indicating that the object was not recognized were recoded as blank cells and excluded from the computation of both modal name agreement and H-values for that item.

To visualize name agreement, we created scatterplots for each variety, highlighting items with particularly high or low name agreement. For each French variety, we also examined the relation between H-values and lexical frequency in exploratory, variety-specific analyses. Because no strictly equivalent lexical frequency measure was available for both varieties, we used the frequency measure available for each dataset: subjective word frequency ratings for Quebec French (Desrochers & Thompson, 2009) and objective corpus-based frequency for France French (New et al., 2004). These correlations were therefore intended to characterize the relation between H-values and the available frequency measure within each variety, rather than to provide a direct cross-variety comparison of equivalent lexical frequency effects.

To compare the full distributions of H-values across varieties, we used kernel density estimation with a Gaussian kernel, following previous work on cross-linguistic name agreement (Duñabeitia et al., 2018). A common bandwidth was derived from the pooled H-values using the default plug-in method of the density function in R (bw = “nrd0”) and then applied identically to the Quebec French and France French distributions.

To compare H-values between France French and Quebec French, we first conducted two distributional comparisons. Because the Quebec French and France French participant samples were independent, a Mann–Whitney U test was used to assess whether one variety tended to yield globally higher or lower H-values than the other. A two-sample Kolmogorov–Smirnov test was also used to assess whether the overall shapes of the H-value distributions differed between varieties. Because the same set of 237 photographs was used in both varieties, we also conducted complementary item-level analyses treating H-values as paired by stimulus. A paired Wilcoxon signed-rank test was used to compare H-values item by item across varieties, and a Spearman correlation was used to examine whether photographs associated with greater lexical dispersion in one variety also tended to show greater lexical dispersion in the other.

All statistical analyses and figures were produced in R with RStudio, and the analysis scripts are available in the Borealis repository.

## Results

### Name agreement norms within each French variety

Norms for all 237 photographs are available in the Borealis repository, with separate normative tables for France French and Quebec French (Table A and Table B). Both the choice of variables and the organization of these normative tables were inspired by Decuyper et al. (2021), who provided Dutch norms for 1,400 BOSS photographs, with adaptations to the aims of the present study.

For each French variety, the tables report the item number, the modal name for each photograph (Modal_Name; the name produced by the largest number of participants, including atypical responses), modal name agreement as the percentage of non-blank responses corresponding to the modal name (Modal_NA, %), and the H-value for each item (H_Value), as well as word frequency measures drawn from existing databases (subjective word frequency for Quebec French, FREQ_MEAN, Desrochers & Thompson, 2009; objective word frequency for France French, FreqFilms2, New et al., 2004). For each photograph, we further report the number of distinct names (Distinct_Names_NA), the two most frequent alternative names other than the modal name (Alt1_Name and Alt2_Name) together with their percentages based on non-blank responses (Alt1_NA, % and Alt2_NA, %), and the number and percentage of blank responses (Blank_Count and Blank_Percent, %).

Table 1 summarizes these norms for Quebec French and France French, including modal name agreement, H-value, proportion of blank responses, and word frequency for items with available frequency data in each French variety. Table 2 reports the 20 items with the highest H-values and the 20 items with the lowest H-values (*N* = 20 each) in the Quebec French and France French datasets.

**Table 1:** Summary of normative measures for the 237 photographs.


VARIABLE	QUEBEC FRENCH				FRANCE FRENCH			
	
	MEAN	SD	MIN	MAX	MEAN	SD	MIN	MAX

Modal name agreement (%)	83.81	18.58	25	100	83.34	18.38	33.02	100

H-value	0.67	0.66	0	3.12	0.73	0.67	0	3.20

Proportion of blank responses (%)	0.64	1.00	0	12.38	0.78	0.91	0	7.55

Subjective word frequency (172 items) (Desrochers & Thompson, 2009)	4.34	1.07	1.75	6.43				

Objective word frequency (224 items) (New et al., 2004)					29.51	60.79	0	570.3


**Table 2:** Highest and Lowest H-Value Items (N = 20 each) in France French and Quebec French.


FRANCE FRENCH						QUEBEC FRENCH					
	
HIGHEST H-VALUES (*N* = 20)			LOWEST H-VALUES (*N* = 20)			HIGHEST H-VALUES (*N* = 20)			LOWEST H-VALUES (*N* = 20)		
			
ITEM NO.	MODAL NAME	H-VALUE	ITEM NO.	MODAL NAME	H-VALUE	ITEM NO.	MODAL NAME	H-VALUE	ITEM NO.	MODAL NAME	H-VALUE

107	carafe	3.20	113	clé	0	196	ciseau à bois	3.12	6	lit	0

196	lime	2.93	120	parapluie	0	197	boulon	2.85	10	chaise	0

15	verre	2.87	134	tomate	0	179	cor	2.78	21	fourchette	0

3	télévision	2.50	136	oignon	0	107	pichet	2.49	32	jupe	0

179	cor	2.39	144	asperges	0	45	lumière	2.44	48	kangourou	0

35	manteau	2.37	159	moto	0	41	scarabée	2.24	57	zèbre	0

49	raton laveur	2.37	180	accordéon	0	156	patin à roues alignées	2.22	58	vache	0

111	grange	2.21	182	cadenas	0	3	télévision	2.21	62	tigre	0

76	putois	2.21	193	ceinture	0	114	clé à molette	2.15	63	ours	0

1	chaise	2.15	212	pomme	0	231	épingle à linge	2.09	64	renard	0

45	feu vert	2.14	216	fraise	0	38	chandail	2.05	68	cheval	0

75	léopard	2.14	217	citron	0	115	fil	2.03	70	girafe	0

53	coquillage	2.10	219	banane	0	184	chemise	2.02	74	lion	0

184	veste	2.04	220	ananas	0	47	index	1.85	113	clé	0

85	barrière	2.00	39	robe	0.08	206	fusil	1.84	123	couronne	0

115	fil	1.92	56	écureuil	0.08	165	oie	1.84	125	cigare	0

166	pingouin	1.92	57	zèbre	0.08	137	maïs	1.83	130	fourmi	0

22	cuisinière	1.91	70	girafe	0.08	110	lime à ongles	1.79	134	tomate	0

175	tambour	1.75	92	nuage	0.08	19	poêle	1.77	136	oignon	0

172	aigle	1.73	105	puits	0.08	95	pain	1.75	139	céleri	0


**Figure 1:**
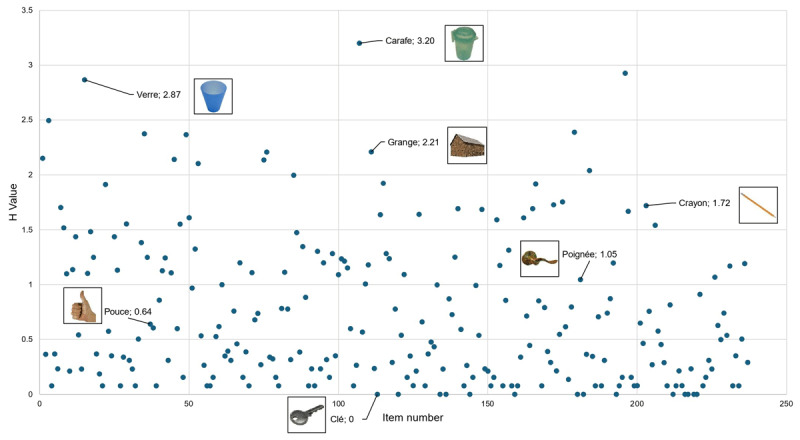
H-values for the 237 photographs in the France French dataset. Each point represents one item, with selected examples annotated using the corresponding photograph, modal name, and H-value.

For the France French data, name agreement results are illustrated in a scatterplot (see Figure 1), which displays H-values for all 237 photographs (x-axis: item number; y-axis: H-value). H-values ranged from 0, indicating no response dispersion and maximal name agreement (e.g., for the picture of a *key*), to 3.20 for the picture of a *carafe*, which showed the greatest diversity of naming responses. The mean H-value in this variety was 0.73 (see Table 1). Fourteen items (5.91%) had an H-value of 0, whereas 115 items (48.52%) had H-values below 0.5, indicating convergent naming for roughly half of the set. Mean modal name agreement was 83.34% (see Table 1), consistent with the fact that most items elicited a clear dominant name. As an exploratory, variety-specific analysis, we examined the relation between H-values and objective corpus-based frequency in France French. For the subset of 224 items with available objective frequency norms (FreqFilms2; New et al., 2004), the Pearson correlation was not significant, *r* = –.004, *p* = .953, 95% CI [–.135, .127], indicating no linear relationship between H-values and objective word frequency in France French.

**Figure 2:**
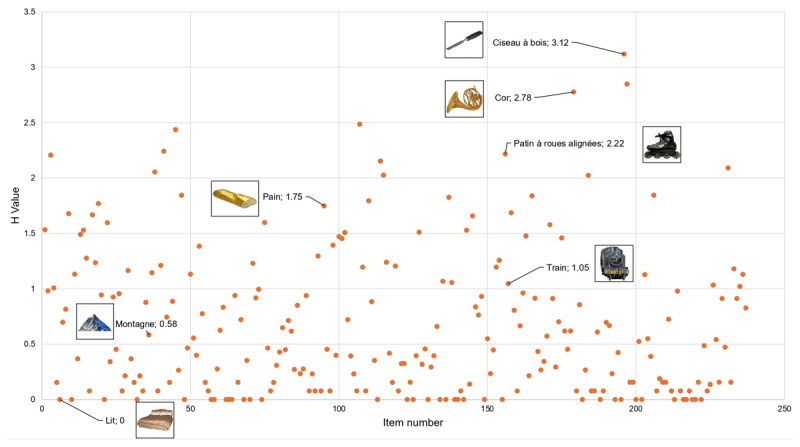
H-values for the 237 photographs in the Quebec French dataset. Each point represents one item, with selected examples annotated using the corresponding photograph, modal name, and H-value.

For the Quebec French data, name agreement results are presented in a similar scatterplot (see Figure 2), which displays H-values for all 237 photographs. H-values ranged from 0, indicating no response dispersion and maximal name agreement (e.g., for the picture of a *bed*) to 3.12 for the picture of a *wood chisel*, which showed the greatest diversity of naming responses. The mean H-value in this variety was 0.67 (see Table 1). Thirty-six items (15.19%) had an H-value of 0, and 124 items (52.32%) had H-values below 0.5, suggesting that more than half of the items elicited convergent naming. Mean modal name agreement was 83.81% (see Table 1), indicating that most items elicited a clear dominant name in Quebec French as well. As an exploratory, variety-specific analysis, we examined the relation between H-values and subjective word frequency ratings in Quebec French. For the subset of 172 items with available subjective frequency ratings (FREQ_MEAN; Desrochers & Thompson, 2009), the Pearson correlation was small but significant, *r* = –.194, *p* = .011, 95% CI [–.334, –.046], indicating that items rated as more frequent tended to show slightly lower H-values, and therefore more convergent naming, in Quebec French. In cases where the modal name was in the plural form, but the corresponding frequency norm was available only for the singular form, we used the singular entry for comparison, because pluralization resulted from our data processing procedure rather than a distinct lexical item (see Supplementary File A).

### Cross-cultural comparison of H-values

**Figure 3:**
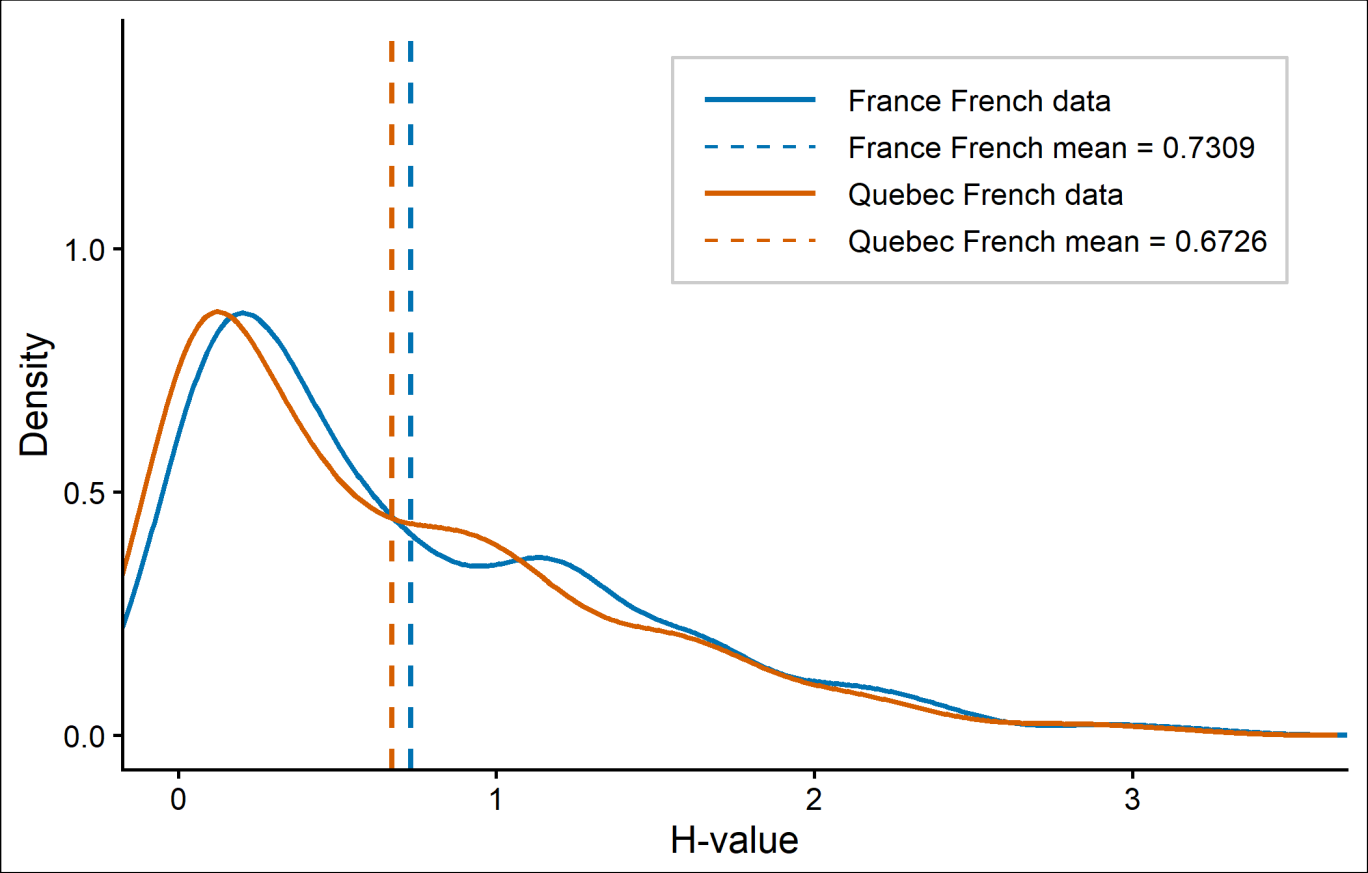
Kernel density estimates of H-value distributions in the France French and Quebec French datasets. Dashed vertical lines indicate the mean H-value for each variety.

To compare name agreement across varieties, we examined the distribution of H-values in France French and Quebec French using a kernel density plot (see Figure 3). In this figure, H-value is shown on the x-axis and estimated density on the y-axis. The two density curves overlap closely and display a similar overall shape, with many items clustered at low H-values and a gradual decrease toward higher values. The France French curve is slightly shifted toward higher H-values, which is consistent with the mean H-values reported in Table 1 (0.73 for France French vs. 0.67 for Quebec French).

To test whether this pattern was statistically reliable, we first conducted two distributional comparisons. A Mann–Whitney U test comparing H-values between the two varieties (*N* = 237 items per variety) indicated no statistically significant difference between the France French and Quebec French distributions of H-values (*U* = 29,823, *p* = .24). A two-sample Kolmogorov–Smirnov test likewise did not provide evidence for a difference in the overall distribution of H-values between the two norms (*D* = .093, *p* = .26).

Because the same photographs were used in both varieties, we also conducted complementary item-level analyses treating H-values as paired by stimulus. The paired Wilcoxon signed-rank test indicated a statistically significant but small item-level difference between Quebec French and France French H-values, with H-values tending to be slightly higher in France French than in Quebec French (*V* = 10,618, *p* = .041, *r* = .14). This test was based on 224 non-zero paired differences, as 13 of the 237 photographs had identical H-values across varieties and therefore did not contribute to the signed-rank calculation. In addition, H-values were positively correlated across the two varieties, indicating that photographs associated with greater lexical dispersion in one variety also tended to show greater lexical dispersion in the other (Spearman’s *ρ* = .651, *p* < .001).

Descriptive comparisons of modal names showed substantial overlap in the labels used across varieties. In 204 out of 237 cases (86.08%), the two groups shared the same modal name for a given photograph, whereas the remaining items (13.92%) showed different dominant labels in France French and Quebec French.

## Discussion

This study provides the first cross-cultural normative dataset on name agreement for Quebec French and France French for 237 photographs from the Bank of Standardized Stimuli (BOSS; Brodeur et al., 2010, 2014), collected in two samples of neurologically typical French-speaking adults (105 Quebec French speakers, 106 France French speakers).

### Implications of name agreement findings in Quebec French and France French

For both varieties, the results point to relatively convergent naming patterns, as reflected in low mean H-values and high modal name agreement.

In France French, the mean H-value was 0.73 and mean modal name agreement was 83.34%, indicating that most photographs elicited a clear dominant label. To our knowledge, no name agreement norms for BOSS photographs were previously available in France French. The present dataset therefore fills this gap by providing item-level name agreement norms for a set of BOSS photographs. The mean H-value observed here is very close to those reported for color line drawings in other French samples. Duñabeitia et al. (2018) reported a mean H-value of 0.71 for 750 items in the MultiPic database, and Bonin et al. (2020) reported a mean of 0.72 for 313 items in IMABASE, both based on France French speakers. The similarity between these values and the mean H-value obtained in the present study suggests that the degree of response dispersion, and therefore the level of lexical consensus, for BOSS photographs in France French is comparable to that observed for other normed picture sets.

In Quebec French, the mean H-value was slightly lower (0.67), with a mean modal name agreement of 83.81%, again indicating that most items elicited a dominant name with relatively little competition from alternatives. Picture-naming tests have previously been developed for Quebec French, including name agreement norms for 60 color drawings collected to construct the TDQ-60 (Test de dénomination de Québec – 60 images) and for 30 BOSS items included in the TDQ-30 (Test de dénomination de Québec – 30 images), both based on large samples of Quebec French speakers and reporting modal and alternative names (Macoir et al., 2018; Macoir et al., 2021). Brodeur et al. (2012) provided Quebec French name agreement norms for 480 BOSS photographs based on 30 neurologically healthy adults, and Masson-Trottier et al. (2017) validated 235 photographs with 13 Quebec French speakers for clinical use. Brodeur et al. (2012) reported a mean H-value of 1.03, which differs from the mean of 0.67 observed in the present sample. This discrepancy may reflect a combination of methodological differences, including data processing choices and the larger number of participants per item in the current study. The present norms extend the available resources by increasing the number of respondents for a defined set of 237 photographs and by providing item-level modal name agreement and H-values as complementary measures for characterizing name agreement in Quebec French.

Taken together, these patterns indicate that the 237 BOSS photographs considered here constitute a set of stimuli that elicit relatively consistent naming in both Quebec French and France French. The norms provide researchers and clinicians with detailed information on modal name agreement and H-value-based response dispersion in each variety, which can be used to select items with higher or lower lexical consensus depending on the goals of a given study or assessment.

### Cultural convergence, divergence, and implications

The present results suggest broadly similar patterns of name agreement in Quebec French and France French. The distributions of H-values were very similar across varieties, and the Mann–Whitney U and Kolmogorov–Smirnov tests did not reveal statistically significant distributional differences between them. At the item level, the paired Wilcoxon signed-rank test indicated a statistically significant but small difference, with H-values tending to be slightly higher in France French than in Quebec French. In addition, the Spearman correlation showed a positive association between H-values across varieties, suggesting that photographs associated with greater lexical dispersion in one variety also tended to show greater lexical dispersion in the other.

Descriptive comparisons of modal names reinforce the idea of similarity in naming patterns. In 204 out of 237 cases (86.08%), Quebec French and France French speakers produced the same modal name for a given photograph, suggesting that a large majority of items elicit a similar dominant label across the two varieties. This overlap indicates that, for many stimuli, cross-cultural use of the same photographs may be feasible. At the same time, 13.92% of the items had different modal names across varieties, and both the mean H-values and the paired item-level comparison pointed to slightly greater lexical dispersion in France French than in Quebec French, although the effect was small.

A similar pattern can be observed in MultiPic, which included two Dutch regional variants: Dutch as used in Belgium and Dutch as used in the Netherlands (Duñabeitia et al., 2018). These two variants were provided as separate normative datasets. A descriptive comparison of the modal names reported in these two datasets showed that 119 out of 750 items had different modal names across the two Dutch varieties, corresponding to 15.87% of the dataset. This proportion is slightly higher than, but close to, the 13.92% observed between Quebec French and France French in the present study, further suggesting that national varieties of the same language may show meaningful differences in naming norms.

An exploratory descriptive review of the 33 items with different modal names indicated that these items were distributed across several semantic domains rather than confined to a single category, with the largest groups involving kitchen utensils (*n* = 7) and clothing-related items (*n* = 6). Many divergences appeared to reflect plausible cross-variety lexical differences, as illustrated by pairs such as *casserole/chaudron*, *pull/chandail*, *moufle/mitaine*, *chaussure/soulier*, *pomme de terre/patate*, *pastèque/melon d’eau*, and *bougie/chandelle*, where the France French modal name is listed first and the Quebec French modal name second. Other cases involved more specialized or potentially ambiguous items, such as *lime/ciseau à bois* or *vis/boulon*. Descriptively, these divergent items also showed greater lexical dispersion than items with the same modal name across varieties: mean H-values were 1.31 in France French and 1.37 in Quebec French for divergent items, compared with 0.64 and 0.56 for items with the same modal name across varieties. Mean modal name agreement was also lower for divergent items, with values of 65.29% in France French and 64.58% in Quebec French, compared with 86.25% and 86.93% for items with the same modal name across varieties.

Taken together, these findings indicate that many photographs can likely be used across Quebec French and France French, but that caution is warranted when importing norms from one variety to the other, particularly for items with low name agreement or different modal names across groups. More broadly, this pattern suggests that culture-specific lexical norms can still shape naming behavior in subtle but non-negligible ways, even when speakers share a common base vocabulary.

### Strengths and limitations of the present study

This study delivers the first cross-cultural norms for name agreement in Quebec French and France French for 237 BOSS photographs, derived from two comparable samples of neurologically typical adult speakers (105 in Quebec, 106 in France). A strength of the present study lies in the transparency and reproducibility of the norms. The data processing procedure, the analysis scripts, and the final item-level normative tables are documented and made available in the Borealis repository, which facilitates reuse and critical evaluation of the dataset. Another strength is that name agreement was collected for the same set of 237 photographs in two similarly sized samples of adult speakers of Quebec French (*N* = 105) and France French (*N* = 106), using parallel online procedures. Although these samples are not intended to be fully representative of all speakers of each variety, this design nonetheless provides directly comparable norms across the two groups while keeping the stimuli and task constant.

A further strength of using photographic stimuli is their flexibility for future expansion and cultural adaptation. Compared with line drawings, photographs can be more easily added, replaced, or adapted to specific cultural contexts, provided that appropriate licensing and quality criteria are respected. This flexibility was used in the present study, as 14 replacement photographs were added under Creative Commons licenses when BOSS photographs were unavailable for some concepts.

The scope of the present normative dataset should also be considered in relation to broader picture-naming norming studies. Unlike some larger normative databases, the present study focused primarily on name agreement and response diversity. It did not collect additional psycholinguistic variables such as conceptual familiarity, image agreement, subjective visual complexity, subjective lexical frequency, or naming latencies. These variables would provide a more complete characterization of the stimulus set and could help future studies examine how multiple psycholinguistic dimensions jointly influence naming performance. In particular, collecting subjective lexical frequency ratings in both Quebec French and France French would allow a more directly comparable analysis of the relation between lexical frequency and name agreement across varieties.

The size of the stimulus set should also be considered when interpreting the scope of the present norms. Although the 237 photographs provide a useful cross-variety core set, this number remains modest compared with larger picture-naming norming databases. The stimulus set was designed to correspond as closely as possible to the classic 260-concept set of Snodgrass and Vanderwart (1980), while keeping the online task at a reasonable duration and limiting participant fatigue. Future work could extend these norms to a larger number of BOSS photographs or additional culturally adapted photographs.

Another methodological limitation concerns the presentation order of the photographs. In the first three data collection waves, photographs were presented in a fixed order based on the sequence proposed by Snodgrass and Vanderwart (1980) and organized by semantic category, whereas the fourth wave used a randomized item order for each participant. A fixed semantic order could potentially create a facilitation effect, particularly when semantically related items are presented close to one another. Because the primary outcome of the present study was the naming response produced rather than naming latency, the impact of presentation order on the main measures is likely limited, but an order effect cannot be entirely ruled out. Nevertheless, an exploratory item-level comparison between the two Quebec French waves showed a strong correlation in H-values, suggesting that the relative pattern of lexical dispersion across items was highly similar despite the difference in presentation order (Spearman’s *ρ* = .887, *p* < .001).

Other limitations should also be acknowledged. First, demographic information was more complete for the Quebec French sample than for the France French sample: exact age and gender were available for only 44 France French speakers, and only age ranges were known for the remaining respondents. This lack of precise age information constrains the extent to which age-related patterns can be examined. Second, both groups were recruited online and should be considered convenience samples rather than fully representative of all Quebec French or France French speakers. Although the two samples were similar in size and yielded stable estimates of name agreement for the present purposes, future studies could aim for larger and more stratified samples, for example with respect to age and gender, to increase representativeness. Finally, the frequency-related analyses should be interpreted with caution because the available lexical frequency measures were not directly equivalent across varieties. Objective corpus-based frequency was available for France French, whereas subjective frequency ratings were used for Quebec French. As a result, these analyses should be understood as exploratory and variety-specific, rather than as a direct comparison of equivalent frequency effects across Quebec French and France French.

## Conclusion

This study established cross-cultural name agreement norms for a set of 237 photographs from the Bank of Standardized Stimuli (BOSS; Brodeur et al., 2010, 2014) in two groups of neurologically healthy adult speakers of Quebec French and France French. The results revealed relatively convergent naming patterns in both varieties, with high modal name agreement, low mean H-values, and a similar item-level profile of lexical dispersion across Quebec French and France French, alongside a subset of items that showed different dominant labels or greater lexical dispersion across varieties.

By offering name agreement norms in both varieties, this work makes available a culturally and linguistically adapted set of photographic stimuli for experimental research and clinical applications. In addition, the comparison of H-values and modal names between Quebec French and France French highlights how cultural context may shape lexical choices for some items, even when speakers share a common language. Future studies could extend these norms to a larger set of BOSS photographs and other francophone varieties, code the sources of modal-name differences more systematically, and incorporate additional psycholinguistic variables such as conceptual familiarity, image agreement, subjective visual complexity, subjective lexical frequency, and naming latencies. Such extensions would provide a more complete characterization of the stimulus set and allow future work to examine how name agreement interacts with other variables in experimental tasks and clinical assessments.

## Additional Files

The additional files for this article can be found as follows:

10.5334/joc.509.s1 Supplementary File A. Data Processing Procedure.

10.5334/joc.509.s2 Tables A and B. France French and Quebec French.

## Data Availability

The 237 photographs used in the study, the data processing procedure, the R scripts used for analysis, and the final item-level normative tables are available in the Borealis repository: https://doi.org/10.5683/SP3/VHE78M. The photographic stimuli include both the BOSS photographs and the 14 replacement photographs obtained under Creative Commons licenses. The photographs are organized so that they correspond directly to the item numbers in the normative tables, reducing the risk of stimulus-selection errors by future users. The normative tables are provided separately for France French and Quebec French and include, for each item, the modal name, modal name agreement percentage, H-value, available lexical frequency measure, number of distinct names, two most frequent alternative names with their percentages, and the number and percentage of blank responses. Permission was obtained from Mathieu Brodeur, Ph.D., to use the BOSS images included in the present study and to make them available in the repository under a CC BY license, with appropriate credit. The additional replacement photographs selected from online repositories are also included according to their Creative Commons licenses. For ethical reasons, the raw participant-level data, processed participant-level datasets, and detailed data-processing logs are not publicly available, as participants did not consent to depositing these materials in an institutional repository. However, the final item-level normative tables provide the information needed to use the stimulus set for research and clinical applications. The dataset will also be made available on the website of the Sensorimotricité, Neuroplasticité et Communication laboratory.

## References

[1] Alario, F., & Ferrand, L. (1999). A set of 400 pictures standardized for French: Norms for name agreement, image agreement, familiarity, visual complexity, image variability, and age of acquisition. Behavior Research Methods, 31(3), 531–552. 10.3758/BF03200732 10502875

[2] Alario, F. X., Ferrand, L., Laganaro, M., New, B., Frauenfelder, U. H., & Segui, J. (2004). Predictors of picture naming speed. Behavior Research Methods, Instruments, & Computers: A Journal of the Psychonomic Society, Inc, 36(1), 140–155. 10.3758/bf03195559 15190709

[3] Barry, C., Morrison, C. M., & Ellis, A. W. (1997). Naming the Snodgrass and Vanderwart Pictures: Effects of Age of Acquisition, Frequency, and Name Agreement. The Quarterly Journal of Experimental Psychology Section A, 50(3), 560–585. 10.1080/783663595

[4] Biederman, I., & Ju, G. (1988). Surface versus edge-based determinants of visual recognition. Cognitive Psychology, 20(1), 38–64. 10.1016/0010-0285(88)90024-2 3338267

[5] Bonin, P., Peereman, R., Malardier, N., Méot, A., & Chalard, M. (2003). A new set of 299 pictures for psycholinguistic studies: French norms for name agreement, image agreement, conceptual familiarity, visual complexity, image variability, age of acquisition, and naming latencies. Behavior Research Methods, 35(1), 158–167. 10.3758/BF03195507 12723790

[6] Bonin, P., Poulin-Charronnat, B., Lukowski Duplessy, H., Bard, P., Vinter, A., Ferrand, L., & Méot, A. (2020). IMABASE: A new set of 313 colourised line drawings standardised in French for name agreement, image agreement, conceptual familiarity, age-of-acquisition, and imageability. Quarterly Journal of Experimental Psychology, 73(11), 1862–1878. 10.1177/1747021820932822 32478594

[7] Bradley, M. M., & Lang, P. J. (2007). The International Affective Picture System (IAPS) in the study of emotion and attention. In J. A. Coan & J. J. B. Allen (Eds.), Handbook of emotion elicitation and assessment. Oxford University Press. 10.1093/oso/9780195169157.003.0003

[8] Brodeur, M. B., Dionne-Dostie, E., Montreuil, T., Lepage, M., & Op de Beeck, H. P. (2010). The Bank of Standardized Stimuli (BOSS), a New Set of 480 Normative Photos of Objects to Be Used as Visual Stimuli in Cognitive Research. PLoS ONE, 5(5). 10.1371/journal.pone.0010773 PMC2879426 20532245

[9] Brodeur, M. B., Guérard, K., & Bouras, M. (2014). Bank of standardized stimuli (BOSS) phase II: 930 new normative photos. PLoS ONE, 9(9), e106953. 10.1371/journal.pone.0106953 25211489 PMC4161371

[10] Brodeur, M. B., Kehayia, E., Dion-Lessard, G., Chauret, M., Montreuil, T., Dionne-Dostie, E., & Lepage, M. (2012). The bank of standardized stimuli (BOSS): comparison between French and English norms. Behavior Research Methods, 44(4), 961–970. 10.3758/s13428-011-0184-7 22351613

[11] Brodie, E. E., Wallace, A. M., & Sharrat, B. (1991). Effect of Surface Characteristics and Style of Production on Naming and Verification of Pictorial Stimuli. The American Journal of Psychology, 104(4), 517–545. 10.2307/1422939 1793125

[12] Cycowicz, Y. M., Friedman, D., Rothstein, M., & Snodgrass, J. G. (1997). Picture naming by young children: norms for name agreement, familiarity, and visual complexity. Journal of Experimental Child Psychology, 65(2), 171–237. 10.1006/jecp.1996.2356 9169209

[13] Decuyper, C., Brysbaert, M., Brodeur, M. B., & Meyer, A. S. (2021). Bank of Standardized Stimuli (BOSS): Dutch Names for 1400 Photographs. Journal of Cognition, 4(1), 33. 10.5334/joc.180 34327304 PMC8300580

[14] Desrochers, A., & Thompson, G. L. (2009). Subjective frequency and imageability ratings for 3,600 French nouns. Behavior Research Methods, 41(2), 546–557. 10.3758/BRM.41.2.546 19363197

[15] Duñabeitia, J. A., Crepaldi, D., Meyer, A. S., New, B., Pliatsikas, C., Smolka, E., & Brysbaert, M. (2018). MultiPic: A standardized set of 750 drawings with norms for six European languages. Quarterly Journal of Experimental Psychology, 71(4), 808–816. 10.1080/17470218.2017.1310261 28326995

[16] Fiez, J. A., & Tranel, D. (1997). Standardized stimuli and procedures for investigating the retrieval of lexical and conceptual knowledge for actions. Memory & Cognition, 25(4). 10.3758/BF03201129 9259631

[17] Gouvernement du Québec. (2025). Vitrine linguistique. Office québécois de la langue française. https://vitrinelinguistique.oqlf.gouv.qc.ca/

[18] Heuer, S. (2016). The influence of image characteristics on image recognition: a comparison of photographs and line drawings. Aphasiology, 30(8), 943–961. 10.1080/02687038.2015.1081138

[19] Lachman, R., Shaffer, J. P., & Hennrikus, D. (1974). Language and cognition: Effects of stimulus codability, name-word frequency, and age of acquisition on lexical reaction time. Journal of Verbal Learning and Verbal Behavior, 13(6), 613–625. 10.1016/S0022-5371(74)80049-6

[20] Leder, H. (1999). Matching Person Identity from Facial Line Drawings. Perception, 28(9), 1171–1175. 10.1068/p281171 10694965

[21] Macoir, J., Beaudoin, C., Bluteau, J., Potvin, O., & Wilson, M. A. (2018). TDQ-60 – a color picture-naming test for adults and elderly people: validation and normalization data. Neuropsychology, Development, and Cognition. Section B, Aging, Neuropsychology and Cognition, 25(5), 753–766. 10.1080/13825585.2017.1372355 28853339

[22] Macoir, J., Chagnon, A., Hudon, C., Lavoie, M., & Wilson, M. A. (2021). TDQ-30-A New Color Picture-Naming Test for the Diagnostic of Mild Anomia: Validation and Normative Data in Quebec French Adults and Elderly. Archives of Clinical Neuropsychology: The Official Journal of the National Academy of Neuropsychologists, 36(2), 267–280. 10.1093/arclin/acz048 31792492

[23] Martínez, N., Matute, H., Goikoetxea, E., & Barca, L. (2020). PicPsy: A new bank of 106 photographs and line drawings with written naming norms for Spanish-speaking children and adults. PLoS ONE, 15(9). 10.1371/journal.pone.0238976 PMC7489540 32925930

[24] Masson-Trottier, M., Marcotte, K., Leonard, C., Rochon, E., & Ansaldo, A. I. (2017). French version of the Phonological Component Analysis: Stimuli selection and validation. Frontiers in Human Neuroscience, 11. 10.3389/conf.fnhum.2017.223.00027

[25] New, B., Pallier, C., Brysbaert, M., & Ferrand, L. (2004). Lexique 2: A New French Lexical Database. Behavior Research Methods, Instruments, & Computers, 36(3), 516–524. 10.3758/BF03195598 15641440

[26] Nishimoto, T., Miyawaki, K., Ueda, T., Une, Y., & Takahashi, M. (2005). Japanese normative set of 359 pictures. Behavior Research Methods, 37(3), 398–416. 10.3758/BF03192709 16405135

[27] Randolph, C., Lansing, A. E., Ivnik, R. J., Cullum, C. M., & Hermann, B. P. (1999). Determinants of confrontation naming performance. Archives of Clinical Neuropsychology: The Official Journal of the National Academy of Neuropsychologists, 14(6), 489–496. 10.1093/arclin/14.6.489 14590576

[28] Reinke, K., & Ostiguy, L. (2016). Le français québécois d’aujourd’hui. Walter De Gruyter. 10.1515/9783110349306

[29] Rhodes, G., Brennan, S., & Carey, S. (1987). Identification and ratings of caricatures: Implications for mental representations of faces. Cognitive Psychology, 19(4), 473–497. 10.1016/0010-0285(87)90016-8 3677584

[30] Rossion, B., & Pourtois, G. (2004). Revisiting Snodgrass and Vanderwart’s Object Pictorial Set: The Role of Surface Detail in Basic-Level Object Recognition. Perception, 33(2), 217–236. 10.1068/p5117 15109163

[31] Schwitter, V., Boyer, B., Méot, A., Bonin, P., & Laganaro, M. (2004). French normative data and naming times for action pictures. Behavior Research Methods, 36(3), 564–576. 10.3758/BF03195603 15641445

[32] Shannon, C. E., & Weaver, W. (1949). The mathematical theory of communication. In C. E. Shannon & W. Weaver (Eds.), The mathematical theory of communication. Urbana: University of Illinois Press.

[33] Sirois, M., Kremin, H., & Cohen, H. (2006). Picture-naming norms for Canadian French: Name agreement, familiarity, visual complexity, and age of acquisition. Behavior Research Methods, 38(2), 300–306. 10.3758/BF03192781 16956106

[34] Snodgrass, J. G., & Vanderwart, M. (1980). A standardized set of 260 pictures: Norms for name agreement, image agreement, familiarity, and visual complexity. Journal of Experimental Psychology: Human Learning and Memory, 6(2), 174–215. 10.1037/0278-7393.6.2.174 7373248

[35] Université de Sherbrooke. (n.d.). Le dictionnaire Usito. Université de Sherbrooke. https://www.usherbrooke.ca/usito/

[36] Viggiano, M. P., Vannucci, M., & Righi, S. (2004). A new standardized set of ecological pictures for experimental and clinical research on visual object processing. Cortex; a Journal Devoted to the Study of the Nervous System and Behavior, 40(3). 10.1016/S0010-9452(08)70142-4 15259329

[37] Vitkovitch, M., & Tyrrell, L. (1995). Sources of Disagreement in Object Naming. The Quarterly Journal of Experimental Psychology Section A, 48(4), 822–848. 10.1080/14640749508401419

